# Prevalence and factors associated with probable HIV dementia in an African population: A cross-sectional study of an HIV/AIDS clinic population

**DOI:** 10.1186/1471-244X-13-126

**Published:** 2013-05-03

**Authors:** Juliet Nakku, Eugene Kinyanda, Susan Hoskins

**Affiliations:** 1 Makerere University College of Health Sciences/Butabika National Referral /Teaching Hospital, P.O.Box 24136 Kampala, Uganda; 2 MRC/UVRI Uganda Research Unit on AIDS & Senior EDCTP Fellowship, Kampala, Uganda; 3 MRC Clinical Trials Unit, London, UK

**Keywords:** HIV dementia, Neuro-cognitive impairment, HIV/AIDS, HIV dementia, Stress, Africa

## Abstract

**Background:**

The HIV/AIDS infection is common in sub-Saharan Africa and is associated with psychological and neuro- cognitive impairment. These conditions, however, remain largely unrecognized. In this study we aimed to determine the prevalence of probable HIV dementia (PHD) in an HIV clinic population in Uganda and to delineate the factors associated with such impairment in these HIV positive individuals.

**Methods:**

Six hundred eighty HIV clinic attendees were surveyed in a cross sectional study. PHD was assessed using the International Dementia Scale (IHDS). Standardized measures were also used to assess clinical, psychological, social and demographic variables. Respondents were aged 18 years and above and did not have severe physical or mental health conditions. Multivariate analysis was conducted to identify associations between PHD and various factors.

**Results:**

The prevalence of probable HIV dementia was 64.4%. PHD was significantly associated with increasing stress scores and psychosocial impairment but not with age, BMI, CD4 count, use of HAART, or a diagnosis of depression or alcohol dependence.

**Conclusion:**

The prevalence of probable HIV dementia in an ambulatory adult HIV positive population in Uganda was 64.4%. Increasing stress scores and psychosocial impairment were significant contributing factors. Clinicians need to be aware of this and to make efforts to identify neuro-cognitive impairment. Secondly there is need for more studies to better understand the relationship between PHD and stress in HIV populations so as to inform patient care.

## Background

The Human Immuno Deficiency Virus (HIV) infection that causes the Acquired Immuno-deficiency Syndrome (AIDS) is prevalent in Africa with eighty percent of HIV positive people worldwide living on the continent, despite the continent constituting only 11–12% of the world’s population [1]. Africa shoulders 75% of the world’s burden of AIDs related deaths [1]. Neuro-cognitive impairment (NCI) in HIV/AIDS remains largely unrecognized especially in sub-Saharan Africa mainly because providers lack the expertise to identify it but also because it is not routinely screened for, despite this recommendation in HIV treatment guidelines [2].

Medical conditions such as chronic depression and neuro-cognitive impairment have been associated with impaired cell- mediated immunity and increased mortality among individuals with HIV/AIDS irrespective of their clinical stage, CD4 count or use of highly active anti-retroviral therapy (HAART) [3]. HIV related NCI occurs on a spectrum ranging from, according to Antinori et al. [4], asymptomatic neuropsychological impairment to mild neuro cognitive disorder (cognitive impairment with mild functional impairment) to frank HIV associated Dementia (marked cognitive impairment and marked functional impairment).

In this study we aimed to determine the prevalence of probable HIV dementia (PHD) among an ambulant HIV clinic population in Entebbe, Uganda. In addition we sought to delineate the factors associated with PHD in these HIV positive individuals.

## Methods

### Study design

This was a cross sectional study undertaken at two HIV clinics in the semi-urban district of Entebbe, Uganda. All consenting eligible HIV-infected patients attending the two HIV clinics in the government health facilities of Entebbe District Hospital and Kigungu Health Centre III were continuously enrolled into this study. To be eligible for inclusion in this study an individual had to be registered with the study HIV clinics, be aged 18 years and above, be fluent in English or Luganda (the local language into which the study instruments had been translated) and not be too physically and mentally sick, as determined by the attending clinician, to undertake the interview. Trained psychiatric nurses recruited from Uganda’s national psychiatric referral hospital at Butabika conducted the structured interviews.

### Data collection tools

The data collection tool consisted of structured and standardized modules which were translated into Luganda and back translated to English by two independent bilingual psychologists. Meetings were held with the translators in order to obtain consensus. The tools included a social demographic questionnaire, The international HIV dementia Scale (IHDS) [5], the Mini neuropsychiatric interview (M.I.N.I plus) [6], a negative coping style index [7], a negative life events score index [7],a stress score index [7].

The Socio-demographic questionnaire identified sex, age, marital status, highest educational attainment, religion, and employment status and various social factors such as food insecurity, distance from the HIV clinic and duration of awareness of HIV status. Clinical factors assessed included the most recent CD4 count, body mass index (BMI), probable HIV dementia and psychiatric diagnoses.

The International HIV Dementia Scale (IHDS) [5] scale was used for measuring probable HIV dementia. This is a bed side screening tool for HIV associated dementia. It can be used in a clinic setting and only requires 2–3 minutes to complete. This tool has been validated in South Africa and Uganda and found to have good psychometric properties in African populations with sensitivity of 88% and 80% and specificity of 50% and 55% respectively at a cut off of 10 or less [5,8]. This tool that assesses for memory impairment, motor and psychomotor speed does not require one to be proficient in English and is brief and inexpensive. Even though it is thought not to be ideal, it is one of the best for use across cultures for screening for neuro-cognitive impairment in HIV [5].

The M.I.N.I. neuropsychiatric interview (MINI Plus) [6] is a modular DSM IV based structured interview for the diagnosis of psychiatric disorder. The psychiatric disorder modules used in this study were for major depressive disorder, suicidality, alcohol use disorders, generalized anxiety disorder and post-traumatic stress disorder.

Negative coping style index [7] was constructed from variables of the Mental Adjustment to Cancer Scale (MAC) [9] whose items had been adapted to the local HIV situation [7]. Each of this scale’s 17 questionnaire items is scored on a 4-item likert scale 1= (definitely does not apply to me), 2= (does not apply to me), 3= (applies to me), 4= (definitely applies to me). To score all the questionnaire items so that they are all in the same direction i.e. higher scores reflecting more negative coping style required that questionnaire items (1,4,5,10,11,12, 13, 15, 16) that are cast positively be reverse scored. For instance item 1: ‘*I have been doing things that I believe will improve my health* e.g. *changed my diet*. A total score was generated so that higher scores reflected a more negative coping style. The Cronbach’s α of this scale in this study was 0.58.

Negative life events score index [7] was constructed from items of the adverse life events module of the European Para-suicide Interview Schedule (EPSIS I) [10] that has previously been modified for the Ugandan situation by Kinyanda and colleagues (2005) [11]. For this study respondents were required to report whether they had experienced each of these events in the last 6 months. Items that were selected for inclusion in this study were those that were thought to be relevant to the HIV social situation in Uganda. The negative life events considered in this study looked at bereavement, severe illness and severe interpersonal conflict in the following significant social relationships; parent, sibling, spouse/lover and child(ren). The individual related negative life events considered in this study examined for severe sickness, interpersonal conflict, feelings of isolation and abandonment, lack of the basic requirements of food, shelter and medicine. Others included job loss, discrimination and worries about personal finances. A total score was generated to reflect the total number of life events reported. This scale had a Cronbach’s α of 0.82 in this study.

The Stress Score index [7] was constructed by scoring each of the reported negative life events on a 3 point likert scale where respondents were asked the question, ‘*how stressful did you find the event*?’ with possible responses being: 0=(not stressing/minimal stressing), 1=(moderately stressing), 2=(severely stressing). A total score was generated where high scores reflected more stress.

### Statistical analysis

Statistical analysis was undertaken using both SPSS version 9.05 (reliability tests) and STATA. Logistic regression models were used to assess univariate associations between the dependent variable (probable HIV dementia-PHD) and independent variables, grouped in sets of demographic, social, clinical and psychological risk factors, with unadjusted and adjusted odds ratio (adjusted for sex and age) reported. After adjusting within each set of risk factors, those that were associated with PHD at a level of significance of 0.1 were entered into a multivariable model to determine their independent effect on PHD. Factors which remained associated with PHD at p<0.05 were retained in the final model.

### Ethical considerations

The study obtained ethical approval from the Uganda Virus Research Institute’s Science and Ethics Committee, the Uganda National Council of Science and Technology and the London School of Hygiene Ethics Committee. Study participants were invited to consent after being provided with adequate information about the study. Respondents found to have significant psychiatric problems were referred to the psychiatric department at Entebbe district hospital for further assessment and management.

## Results

### Study population

From 6th May 2010 to 10th August 2010, 680 patients attending the HIV clinics at Entebbe hospital and Kigungu health centre III were screened and given appointments to be interviewed for this study. Of these 618 (90.9%) were eventually enrolled into the study while 62 (9.1%) failed to turn up for their interview appointment even after being repeatedly contacted by telephone. Those who refused to participate in this study did not differ significantly from those who were enrolled into the study on gender and age. Of those who were enrolled into the study, Entebbe district hospital contributed 531 (85.9%) while the smaller Kigungu Health Centre III contributed 87 (14.1%).

Like in many HIV clinics in sub-Saharan Africa, the majority of respondents were female (449, 72.7%). Eighty three percent were less than 45 years of age. The population served by the two health centers was similar in terms of gender and age. The majority of respondents (354, 57.3%) had little (less than 8 years) or no formal education. The prevalence of probable HIV dementia as evidenced by a score of ≤10 on the IHDS was 64.4% (95%CI, 60.3%-67.9%).

Two hundred and ninety one (291, 64.8%) of all the female respondents and 105 (62.1%) of the male respondents were positive for PHD. There was no statistically significant difference between the gender on this variable. Age was not associated with PHD nor was marital status nor level of highest educational attainment (Table 1).

**Table 1:** **Socio**-**demographic risk factors by probable HIV dementia** (**PHD**)

	**Number in study (N,%)**	**Probable HIV dementia (n,%)**	**Unadjusted OR (95% CI)**	**Adjusted OR**^**§ **^**(95% CI)**
**Sex**			**P**=**0**.**536**	
Male	169 (27.3)	105 (62.1)	1	
Female	449 (72.7)	291 (64.8)	1.12 (0.78-1.62)	
**Age** (**years**)			**P**=**0**.**927**	
19-24	58 (9.4)	36 (62.1)	1	
25-34	238 (38.6)	150 (63.0)	1.04 (0.58-1.88)	
35-44	217 (35.1)	147 (67.7)	1.28 (0.70-2.34)	
45+	103 (16.7)	62 (60.2)	0.92 (0.48-1.79)	
**Marital status**			**P**=**0**.**669**	**P**=**0**.**592**
Currently married/Cohabiting	275 (48.5)	188 (63.1)	1	1
Widowed	75 (13.2)	54 (65.1)	1.09 (0.65-1.81)	1.12 (0.65-1.94)
Separated/Divorced	132 (23.3)	104 (72.7)	1.56 (1.01-2.41)	1.56 (0.99-2.44)
Single	85 (15.0)	49 (52.7)	0.65 (0.41-1.04)	0.63 (0.39-1.02)
**Education level**			**P**=**0**.**276**	**P**=**0**.**246**
No education	65 (10.5)	45 (69.2)	1	1
Primary only	289 (46.8)	187 (64.7)	0.81 (0.46-1.45)	0.84 (0.47-1.50)
Secondary and Above	264 (42.7)	164 (62.1)	0.73 (0.41-1.30)	0.73 (0.40-1.31)
**Religion**			**P**=**0**.**081**	**P**=**0**.**167**
Christians	535 (86.9)	336 (62.8)	1	1
Moslems	81 (13.1)	59 (72.8)	1.59 (0.94-2.67)	1.60 (0.94-2.70)
**Employment Status**			**P**=**0**.**009**	**P**=**0**.**006**
Farmer/Fisherman	97 (15.7)	67 (69.1)	1	1
Professional/Clerical	43 (7.0)	29 (67.4)	0.93 (0.43-2.00)	0.87 (0.41-1.90)
Tradesperson/artisan/transport worker	262 (42.4)	174 (66.4)	0.88 (0.54-1.46)	0.86 (0.51-1.44)
Unemployed/house Wife	131 (21.2)	85 (64.9)	0.83 (0.47-1.45)	0.78 (0.43-1.42)
Others (including students)	84 (13.6)	40 (47.6)	0.41 (0.22-0.75)	0.40 (0.21-0.73)

Note:^§^ Adjusted for age and sex.

Table 2, eight percent (50, 8%) of our respondents had a MINI-plus diagnosable major depressive disorder. The majority of these, (30, 60.6%) had PHD i.e. scored ≤10 on the IHDS. A diagnosis of common mental disorder including major depressive disorder, alcohol dependence, generalized anxiety disorder and post traumatic stress disorder did not show association with PHD.

**Table 2:** **Clinical risk factors by probable HIV dementia** (**PHD**)

**Clinical condition**	**Number in study (N,%)**	**Probable HIV dementia (n,%)**	**Unadjusted OR (95% CI)**	**Adjusted OR**^**§ **^**(95% CI)**
**Diagnosis of post traumatic stress disorder**			**P**=**0**.**303**	**P**=**0**.**302**
Present	10 (1.6)	8 (80.0)	2.27 (0.48-10.8)	2.28 (0.48-10.91)
**Diagnosis of generalized anxiety disorder**			**P=0.166**	**P=0.5730**
Present	5 (0.81)	5 (100)	**P=0.559**	**P= 0.5309**
**Alcohol dependency disorder**			**P**=**0**.**561**	**P**=**0**.**573**
Present	4 (0.65)	2 (50.0)	0.56 (0.08-3.99)	0.56 (0.08-4.11)
**Major depressive disorder**			**P**=**0**.**531**	**P**=**0**.**514**
Present	50 (8.1)	30 (60.0)	0.83 (0.46-1.49)	0.82 (0.45-1.49)
**Most Recent CD4 count** (**cells**/μ**L**)			**P**=**0**.**088**	**P**=**0**.**061**
<100	66 (12.5)	52 (78.8)	1	1
100-249	159 (30.2)	105 (66.0)	0.52 (0.27-1.03)	0.52 (0.26-1.03)
250-349	81 (15.4)	46 (56.8)	0.35 (0.17-0.74)	0.34 (0.16-0.72)
350+	220 (35.6)	142 (64.6)	0.49 (0.26-0.94)	0.47 (0.24-0.91)
**BMI Index**			**P**=**0**.**205**	**P**=**0**.**166**
Underweight	56 (9.2)	32 (57.1)	1	1
Normal	388 (64.9)	262 (67.5)	1.56 (0.88-2.76)	1.58 (0.88-2.81)
Overweight	117 (20.0)	65 (55.6)	0.94 (0.49-1.78)	0.93 (0.48-1.80)
Obese	35 (5.9)	20 (57.1)	1.00 (0.42-2.35)	0.93 (0.38-2.24)
**ON HAART**			**P**=**0**.**559**	**P**=**0**.**5309**
Yes	399 (64.6)	259 (64.1)	1.11 (0.79-1.56)	1.09 (0.76-1.58)
No	219 (35.4)	137 (62.6)	1	1

Note:^§^ Adjusted for age and sex.

About two thirds (399, 64.6%) of all respondents were on HAART with the majority (456, 75.2%) having been on HAART for more than 12 months. Of these 64.1% scored ≤10 on the IHDS signifying PHD. However, there was no statistical significant association between PHD and being on HAART. The median CD4 count in this study was 300cells/μL. Of the two clinical parameters BMI and recent CD4 count (last 6 months), only recent CD4 count almost attained a significant association with PHD (Table 3).

**Table 3:** **Psychosocial risk factors by probable HIV dementia** (**PHD**)

	**Number in study (N,%)**	**Probable HIV dementia (n,%)**	**Unadjusted OR (95% CI)**	**Adjusted OR**^**§ **^**(95% CI)**
**Social Support**			**P**=**0**.**644**	**P**=**0**.**179**
Low	188 (30.4)	123 (65.4)	1.09 (0.76-1.56)	1.1. (0.76-1.59)
High	430 (69.6)	273 (63.5)	1	1
**Negative life events**			**P**<**0**.**001**	**P**<**0**.**001**
1-5 events	350 (56.6)	199 (56.9)	1	1
6-10 events	191 (43.7)	138 (72.8)	2.03 (1.38-2.97)	2.14 (1.45-3.15)
11 +	77 (12.5)	58 (74.3)	2.32 (1.32-4.05)	2.35 (1.33-4.13)
**Stress Score index**			**P**<**0**.**001**	**P**<**0**.**001**
Low (score 0)	164 (26.5)	77 (46.9)	1	1
Medium (score 1–10)	323 (52.3)	222 (68.7)	2.48 (1.69-3.65)	2.55 (1.73-3.77)
High (score>10)	131 (21.2)	97 (74.0)	3.22 (1.96-5.30)	3.29 (1.99-5.45)
**Negative Coping Style index**			**P**=**0**.**554**	**P**=**0**.**606**
Low	101 (16.3)	81 (80.2)	1	1
Medium	322 (52.1)	160 (52.5)	0.27 (0.16-0.47)	0.26 (0.15-0.44)
High	195 (31.6)	146 (74.9)	0.74 (0.41-1.32)	0.71 (0.39-1.28)
**Psychosocial Impairment**			**P**=<**0**.**001**	**P**=<**0**.**001**
Present	318 (51.5)	176 (55.3)	0.45 (0.32-0.63)	0.45 (0.32-0.64)

Note: ^§^ Adjusted for age and sex.

Of the psychosocial factors assessed the following were associated with PHD: an increasing number of negative life events i.e. 6–10 events (aOR=2.14; 95% CI= 1.45-3.15); and 11+ events (aOR =2.35, 95% CI= 1.33-4.13) as compared to having 1–5 negative life events. Increasing stress scores i.e.medium stress scores (aOR=2.55; 95% CI 1.73-3.77); and high stress scores (aOR=3.29, 95% CI=1.99-5.45) as compared to having low stress scores. Psychosocial impairment was significantly associated with PHD (aOR=0.45, 95% CI= 0.32-0.64) but not negative coping style.

### Factors associated with PHD at multivariate analysis

When a multivariate analysis model was constructed, only the stress score index and psychosocial impairment remained significantly associated with PHD (Table 4). None of the demographic characteristics or clinical factors studied (either psychiatric or physical) was significantly associated with PHD at multivariate analysis.

**Table 4:** Final multivariate model of risk factors for probable HIV dementia

	**(95% CI)**
**Negative life events**	**P**=**0**.**344**
1-5 events	1
6-10 events	1.45 (0.88-2.40)
11 +	1.95 (0.78-4.86)
**Stress Score index**	**P**=**0**.**013**
Score 0	1
Score 1-10	1.99 (1.28-3.09)
Score >10	1.83 (0.80-4.16)
**Psychosocial Impairment**	**P**<**0**.**001**
Present	0.43 (0.31-0.61)

## Discussion

Although the prevalence of PHD in HIV has been reported to range from 12% - 56% using a range of neuropsychiatric batteries [12-14], sub-Saharan Africa contributes very little to this body of knowledge. This study attempts to address this problem. Nakasujja et al. [15] found a prevalence of IHDS identified PHD to be 68.6% in a cohort of highly selected HIV+ adults not yet started on HAART. In our study the prevalence of IHDS identified PHD was very similar (64.4%) in an adult HIV clinic population the majority of whom (75.2%) were on HAART. This underlines the absence of association between use of HAART and IHDS identified PHD in HIV/AIDS individuals in Uganda. This probably points to the fact that HAART in Uganda like in many other sub-Saharan African settings is initiated late or that the regimens used have poor penetration of the blood brain barrier and therefore low capacity to prevent the occurrence of HIV dementia.

### Social demographic correlates

The association between PHD and advanced age has been described before [12-16]^.^ In this study this association between PHD and age was not demonstrated. Ordinarily neuro-cognitive performance declines with increasing age even without the additional risk of HIV. This decline however is known to occur beyond age 50 years. In our study the majority of the respondents were below 45 years of age (83.3%) which may have been the reason for the absence of association between older age and PHD. The lack of association between PHD and education level confirms the suitability of the constructs within the IHDS which are not dependent on the educational status of an individual.

### Clinical correlates

The rate of depression symptoms among individuals with PHD in Ugandan HIV+ adult populations is high [15]. Depression presents with cognitive symptoms such as concentration and memory difficulties [17] but also has been reported to be the first sign of neuro-cognitive impairment or to make NCI worse [13,18]. Our study did not show any association between PHD and major depressive disorder. Similar findings have been reported by other researchers [19]^.^

In this study PHD was only marginally associated with recent CD4 count at bivariate analysis. A significant association between recent CD4 count and PHD has previously been demonstrated by earlier researchers [12,13]. Usually a low CD 4 count is used as a marker of HIV disease progression and this has previously been found to be associated with declining cognitive performance. The relationship between PHD and most recent CD4 count is however, is not a fore gone conclusion as some studies have not reported this association [16]. The variability of results in this area could be attributed to many factors including differences in the elements of NCI measured, the severity of impairment i.e. HIV associated dementia as opposed to mild neuro- cognitive disorder or both and in the variety of measurement tools.

### Psychosocial correlates

It has been argued that stress, coping, social support and life events may be related to HIV disease progression [19]. There are however few studies into the association of these factors with neuro-cognitive impairment in HIV [20]. This study is one of the few that demonstrates this association. It would be tempting to attribute the association between stress and PHD to advanced HIV disease. However, the lack of a significant association between PHD and CD4 count in this study (a marker of HIV disease progression) points to a more complex relationship between these variables and PHD which this study was not designed to elucidate. Further research into this area is therefore required.

### Limitations of this study

The International HIV Dementia Scale used to measure PHD in this study is a screening tool for HIV dementia not a diagnostic assessment tool. For this reason, in this study we report on ‘probable HIV dementia’ (PHD) and not on a diagnosis of ‘HIV dementia’. However this tool has previously been validated to screen for HIV dementia in the Ugandan situation with good psychometric properties observed during the validation study [5].

## Conclusion

The prevalence of PHD in an ambulatory adult HIV+ population in Uganda obtained by the IHDS was 64.4%. Gender, age, CD4 count, use of HAART and a diagnosis of common mental disorder including major depressive disorder were not associated with PHD on multivariate analysis. Only a high stress score index and psychosocial impairment showed such an association. Clinicians need to be aware of the high risk of PHD among HIV patients and to make efforts to objectively and appropriately diagnose and manage it. Secondly there is need for more studies to better understand the relationship between PHD and stress in HIV populations so as to inform patient care.

## Abbreviations

AIDS: Acquired immune deficiency syndrome; BMI: Body mass index; HAART: Highly active anti retroviral therapy; HAD: HIV associated dementia; HIV: Human Immune deficiency virus; IHDS: International HIV Dementia Scale; NCI: Neuro- cognitive impairment; PHD: Probable HIV dementia.

## Competing interests

We declare that there are no competing interests, financial or non-financial.

## Authors’ contributions

JN contributed significantly to the design of the study, acquisition and interpretation of data, drafting and revising of the manuscript for publication. EK was significantly involved in the conception, design and analysis of the data. He also contributed to the drafting and revision of the manuscript and gave final approval to its publication. SH was significantly involved in the conception and design of the study as well as the revision of the manuscript and final approval of its publication.

## Pre-publication history

The pre-publication history for this paper can be accessed here:

http://www.biomedcentral.com/1471-244X/13/126/prepub
